# Ropivacaine inhibits proliferation, migration, and invasion while inducing apoptosis of glioma cells by regulating the SNHG16/miR-424-5p axis

**DOI:** 10.1515/biol-2020-0108

**Published:** 2020-12-31

**Authors:** Rong Liu, Min Wu, Guiju Xu, Lu Ju, Jinhui Xiao, Wei Zhong, Xiao He, Yan Yang

**Affiliations:** Department of Anesthesiology, The 908th Hospital of Chinese PLA Logistical Support Force, No.4, Hudong Road, Yuehu District, Yingtan 335000, Jiangxi, China; Department of Internal Medicine, Ruijin Hospital of traditional Chinese Medicine, Ruijin, Jiangxi, 342500, China; Department of Hepatobiliary Surgery, The First Affiliated Hospital of Gannan Medical University, No.128, Jinling Road, Golden Development Zone, Ganzhou, Jiangxi, 341000, China; Department of Anesthesiology, Ruijin Maternal and Child Health Hospital, Ruijin, Jiangxi, 342500, China

**Keywords:** ropivacaine, lncRNA, SNHG16, miR-424-5p, glioma

## Abstract

**Background:**

Regional anesthesia has anti-proliferative and pro-apoptotic effects in various cancers. Therefore, the purpose of this study was to investigate the effects of ropivacaine on the proliferation, migration, invasion, and apoptosis of glioma cells *in vitro*.

**Methods:**

Under ropivacaine stimulation conditions, proliferation, apoptosis, migration, and invasion of glioma cells were determined by 3-(4,5-dimethylthiazol-2-yl)-2,5-diphenyl-2*H*-tetrazol-3-ium bromide (MTT), flow cytometry, and transwell assays, respectively. Western blot assay was employed to measure the protein expression levels in glioma cells. The expression levels of small nucleolar RNA host gene 16 (SNHG16) and miR-424-5p were assessed by reverse transcription-quantitative polymerase chain reaction (RT-qPCR). The interaction relationship between SNHG16 and miR-424-5p was predicted and confirmed using a bioinformatics database and dual-luciferase reporter, RNA immunoprecipitation (RIP) and RNA pull-down assays.

**Results:**

After treatment with ropivacaine, proliferation, migration, and invasion were repressed while apoptosis was enhanced in glioma cells in a dose-depended manner. In addition, ropivacaine impeded SNHG16 expression in glioma cells. Importantly, overexpression of SNHG16 abolished the ropivacaine-induced effects on glioma cells. Analogously, knockdown of miR-424-5p counteracted the function of ropivacaine in glioma cells. We also found that SNHG16 bound to miR-424-5p and negatively regulated miR-424-5p expression in glioma cells. The rescue experiments indicated that ropivacaine might regulate glioma progression by targeting the SNHG16/miR-424-5p axis.

**Conclusion:**

Our findings revealed the anti-tumor effects of ropivacaine in glioma by targeting the SNHG16/miR-424-5p axis. These data might extend the understanding of regulatory mechanisms by which ropivacaine could suppress glioma development.

## 1 Introduction

Glioma is a common malignant tumor in the central nervous system, constituting the majority of primary malignant brain tumors [1]. Glioma therapeutic treatments include surgical resection, radiotherapy, and chemotherapy; however, the clinical outcomes for patients with glioma remain unsatisfactory [2,3], especially for patients with high-grade glioma [4].

Long noncoding RNAs (lncRNAs), more than 200 nucleotides in length, are non-protein coding transcripts [5]. Importantly, the dysregulation of lncRNA has been reported to participate in the development of various cancer types and could therefore function as a biomarker for cancer diagnosis and prognosis [6]. Furthermore, several lncRNAs were found to be closely associated with the development of glioma. For instance, Zhou et al. reported that lncRNA SPRY4-IT1 was highly connected with glioma grade and tumor size, implying that lncRNA SPRY4-IT1 had huge potential for clinical application [7]. Moreover, the elevated expression of small nucleolar RNA host gene 16 (SNHG16) was found in colorectal cancer and tightly implicated in poor prognosis of patients [8]. Importantly, gain-of-function studies confirmed that overexpression of SNHG16 promoted breast cancer cell migration [9]. Conversely, knockdown of SNHG16 reduced cell viability, induced apoptotic cell death, and impeded cell migration [10]. Thus, we predicted that SNHG16 might play a key role in glioma.

Recently, dysregulation of miRNA has been considered to act as a key function in the pathogenesis and development of human diseases, particularly cancers [11]. Notably, miRNAs could function as crucial modulators of gene expression via interaction with argonaute protein, thereby forming the RNA-induced silencing complex to inhibit expression of target genes [12]. In addition, miR-424-5p has been reported to exert anti-tumor functions in multiple malignancies [13,14,15]. Gao et al. indicated that miR-424 was reversely regulated by the cancerogenic gene plasmacytoma variant translocation 1 (PVT1) in cervical cancer [16]. It is possible that miR-424 also acted as a carcinoma inhibitor in glioma [17]; however, the biological role and regulatory mechanism of miR-424 in glioma were less clear.

In this paper, we inspected the function of ropivacaine on glioma cell growth, apoptosis, and mobility. We further showed the relationship between SNHG16 and miR-424-5p by performing bioinformatics database analysis and functional experiments in glioma.

## 2 Materials and methods

### 2.1 Cell culture

Glioma cell lines (T98G and LN229) were purchased from the American Type Culture Collection (Rockville, MD, USA). The cells were propagated in RPMI medium 1640 (Hyclone, Logan, UT, USA) containing 10% (v/v) fetal bovine serum (FBS; Biochrom KG, Berlin, Germany) and 1% penicillin/streptomycin (Biochrom KG) under standard culture conditions (5% CO_2_, 37°C). To establish ropivacaine (AstraZeneca, London, UK) stimulation for cell culture, T98G and LN229 cells were cultured with different doses of ropivacaine (0, 0.25, 0.5, or 1 mM) over three time periods (24, 48, or 72 h).

### 2.2 3-(4, 5-Dimethylthiazol-2-yl)-2,5-diphenyl-2*H*-tetrazol-3-ium bromide (MTT) assay

T98G and LN229 cells were seeded into 96-well plates at a concentration of 3,000 cells per well. MTT assay was performed to examine the viability of T98G and LN229 cells according to the manufacturer’s instructions. Following incubation for 24, 48, and 72 h, 20 µL of MTT solution (Sigma, Louis, Missouri, USA) was added to each well and incubated at 37°C. After 4 h, 150 µL of dimethyl sulfoxide (DMSO) was used to dissolve formazan crystals in the 96-well plates. A microplate reader (BioTek Instruments, Winooski, VT, USA) was utilized to evaluate optical density at a wavelength of 490 nm in each well of the plate.

### 2.3 Cell apoptosis assay

The annexin V-fluorescein isothiocyanate (FITC)/propidium iodide (PI) kit (Sigma) was used to detect cell apoptosis. Briefly, T98G or LN229 cells were collected at 48 h after transfection and then resuspended with phosphate buffer at a concentration of 1 × 10^6^/mL. About 10 µL of staining solution, including annexin V labeled with FITC and PI, was dropped into each sample under dark conditions and incubated for 30 min. A flow cytometer (Thermo Fisher Scientific, Waltham, MA, USA) and Cell Quest software were used to quantify and analyze the apoptosis rate, respectively.

### 2.4 Transwell assay

A transwell assay was performed for migration and invasion analyses. The transwell inserts were pre-adhered with or without Matrigel (BD Biosciences, San Jose, CA, USA) and a total of 300 µL of cell suspension in serum-free medium (5 × 10^6^ cells per mL) was added into the upper chambers. The bottom chamber had medium containing 10% FBS added to induce cell invasion and migration. After culturing for 24 h, invaded or migrated cells were fastened with 4% formaldehyde and then dyed with 0.1% crystal violet. The invaded or migrated cells were photographed and counted under a light microscope (Nikon, Tokyo, Japan).

### 2.5 Western blot assay

Total protein was extracted using radioimmunoprecipitation assay (RIPA) buffer (Solarbio, Beijing, China). Equal protein for each sample was separated by 12% sodium dodecyl sulfate polyacrylamide gel electrophoresis and then electrophoretically transferred to polyvinylidene fluoride membranes (GE Healthcare, Piscataway, NJ, USA). Subsequently, membranes were blocked with 5% skim milk solution at room temperature for 1 h. Protein expression was detected through incubation of the membranes with indicated primary antibody at 4°C overnight. After being washed three times for 10 min each, membranes were incubated with Goat polyclonal Secondary Antibody to Rabbit IgG-H&L (ab1500771; 1:2,000 dilution; Abcam, Cambridge, MA, USA) for 1 h at room temperature. ECL Western Blotting Detection Kit (Solarbio) and Image J software (National Institutes of Health, Bethesda, MD, USA) were used to visualize and quantify protein expression, correspondingly. The primary antibodies used were proliferating cell nuclear antigen (PCNA; ab92552; 1:1,000 dilution; Abcam), Cleaved-caspase-3 (Cleaved-cas 3; ab2302; 1:1,000 dilution; Abcam), matrix metalloproteinase 2 (MMP-2; ab97779; 1:1,000 dilution; Abcam), and glyceraldehyde-3-phosphate dehydrogenase (GAPDH; ab181602; 1:3,000 dilution; Abcam).

### 2.6 RNA isolation and reverse transcription-quantitative polymerase chain reaction (RT-qPCR)

An RT-qPCR assay was implemented to evaluate the expression levels of SNHG16 and miR-424-5p in cells. Total RNA was isolated using Trizol reagent (Thermo Fisher Scientific) referring to the manufacturer’s procedures. After that, a TaqMan Reverse Transcription Kit (Applied Biosystems, Carlsbad, CA, USA) or TransScript miRNA First-Strand cDNA Synthesis SuperMix (Transgen biotech, Beijing, China) was applied to convert RNA to complementary DNA (cDNA). The RT-qPCR assay was performed with 2 µL of cDNA, primers, SYBR-Green buffer, and nuclease-free water in 10 µL of reaction mixture in BIO-RAD IQTM5 Multicolor Real-Time PCR detection system (Bio-Rad, Hercules, CA, USA), followed by 95°C for 15 s and 60°C for 1 min for 40 cycles. The transcript levels of SNHG16 and miR-424-5p were evaluated based on the 2^−ΔΔCt^ method and standardized to GAPDH and endogenous small nuclear RNA U6, individually.

The sequences of primers were as follows:

SNHG16 (F, 5′-CAGAATGCCATGGTTTCCCC-3′; R, 5′-TGGCAAGAGACTTCCTGAGG-3′);
miR-424-5p (F, 5′-GCCGAGCAGCAGCAATTCATGT-3′; R, 5′- CTCAACTGGTGTCGTGGA-3′);
GAPDH (F, 5′-TCCCATCACCATCTTCCAGG-3′; R, 5′- GATGACCCTTTTGGCTCCC-3′);
U6 (F, 5′-AACGCTTCACGAATTTGCGT-3′; R, 5′- CTCGCTTCGGCAGCACA-3′).

### 2.7 Transfection assay

SNHG16-overexpression vector (SNHG16) and its negative control (Vector), specific small interfering RNA (siRNA) against SNHG16 (si-SNHG16) and siRNA scrambled control (si-NC), miR-424-5p mimic and miR-NC, Anti-miR-424-5p, and Anti-NC were purchased from RiboBio (Guangzhou, China). Construction of SNHG16-overexpression vector (SNHG16) was accomplished by amplifying cDNA of SNHG16 and subcloning into the pcDNA vector (RiboBio). The vectors or oligonucleotides were transfected into T98G or LN229 cells by Lipofectamine 2000 (Thermo Fisher Scientific) in compliance with the manufacturer’s protocol. The primers for SNHG16-overexpression were F 5′-aagcttGCGTTCTTTCGAGG-3′ and R 5′-ggatccTGACGGTAGTTTCCC-3′, including HindIII and BamHI restriction sites. The sequence for si-SNHG16 was 5′-UAAAGACAUGGCACUUUGGGU-3′, and the sequence for si-NC was 5′-UUCUCCGAACGUGUCACGUTT-3′.

### 2.8 Dual-luciferase reporter assay

StarBase (http://starbase.sysu.edu.cn/) was used to predict the target gene of SNHG16. The fragments of the SNHG16, containing an interaction site with miR-424-5p, were amplified from cDNA and introduced into the restriction sites (BamHI and NotI) of Dual-Luciferase reporter pRL-TKn (Realgene, Nanjing, China), named as SNHG16-WT. The Mut Express II Fast Mutagenesis Kit (Vazyme, Nanjing, China) was used to create mutations of the binding site. Afterward, the sequence was also inserted into a plasmid, named as SNHG16-MUT. T98G and LN229 cells were co-transfected with 50 nM of miR-424-5p mimic or miR-NC and 0.4 µg of SNHG16-WT or SNHG16-MUT with Lipofectamine 2000 (Thermo Fisher Scientific). After 48 h, the firefly luciferase signal was detected using a Dual-Luciferase Reporter assay system (Promega, Madison, WI, USA) and normalized to the Renilla luciferase signal.

### 2.9 RNA immunoprecipitation (RIP) assay

RIP assay was performed using the RIPTM RNA binding Protein Immunoprecipitation kit (Sigma). In short, T98G and LN229 cells were lysed in complete RIP lysis buffer. Furthermore, pro-covered mouse IgG or Anti-Ago2 antibody (Sigma) magnetic beads were added into cell lysates for overnight incubation. The immunoprecipitated RNA was extracted and then subjected to a RT-qPCR assay to detect the expression levels of SNHG16 and miR-424-5p.

### 2.10 RNA pull-down assay

T98G and LN229 cells were harvested and lysed with 0.7 mL of lysis buffer (5 mM MgCl_2_, 100 mM KCl, 20 mM Tris [pH 7.5], 0.3% NP-40, 50 U of RNase inhibitor; Invitrogen, Carlsbad, CA, USA). The cell lysates were incubated with the biotin-labeled miR-424-5p probe (Bio-miR-424-5p) synthesized by RiboBio. After the biotin-coupled RNA complex was pulled down by streptavidin-coated magnetic beads, the RNA complexes adsorbed the beads and were eluted and purified with lysis buffer and proteinase K, respectively. The abundance of SNHG16 in the bound fraction was measured by RT-qPCR analysis.

### 2.11 Statistical analysis

Statistical analyses were performed using GraphPad Prism 7 (GraphPad, La Jolla, CA, USA). The one-way analysis of variance followed by Tukey’s posttest was used to assess statistically significant differences between each group. Data were presented as mean ± standard deviation, and a *P*-value of less than 0.05 was regarded as statistically significant.

## 3 Results

### 3.1 Ropivacaine inhibited proliferation and induced apoptosis of glioma cells

**Figure 1:**
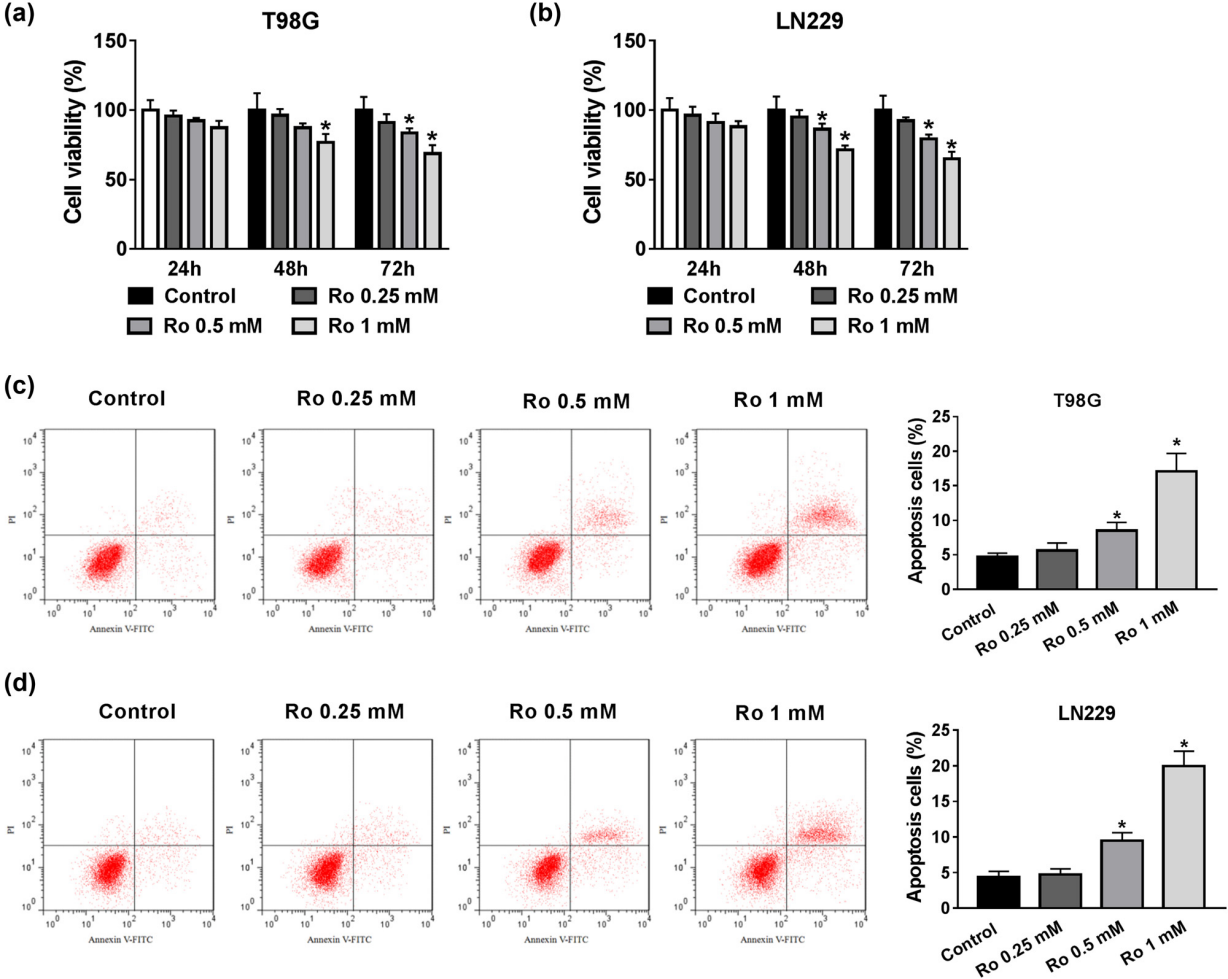
The effect of different concentrations of ropivacaine on cell proliferation and apoptosis of glioma cells. (a and b) The cell viability of T98G and LN229 cells treated with ropivacaine was assessed by MTT assay. (c and d) The flow cytometry assay was used to monitor cell apoptosis in T98G and LN229 cells treated with different concentrations of ropivacaine for 48 h. *P < 0.05.

To identify ropivacaine function involved in proliferation and apoptosis, T98G and LN229 cells were treated with different doses of ropivacaine. Analysis of the MTT assay results indicated that ropivacaine repressed cell proliferation in a time/concentration-dependent manner (Figure 1a and b). Additionally, ropivacaine was found to induce the apoptosis of T98G and LN229 cells in a concentration-dependent manner (Figure 1c and d). Therefore, ropivacaine could repress proliferation and induce apoptosis of glioma cells.

### 3.2 Ropivacaine suppressed migration and invasion of glioma cells

**Figure 2:**
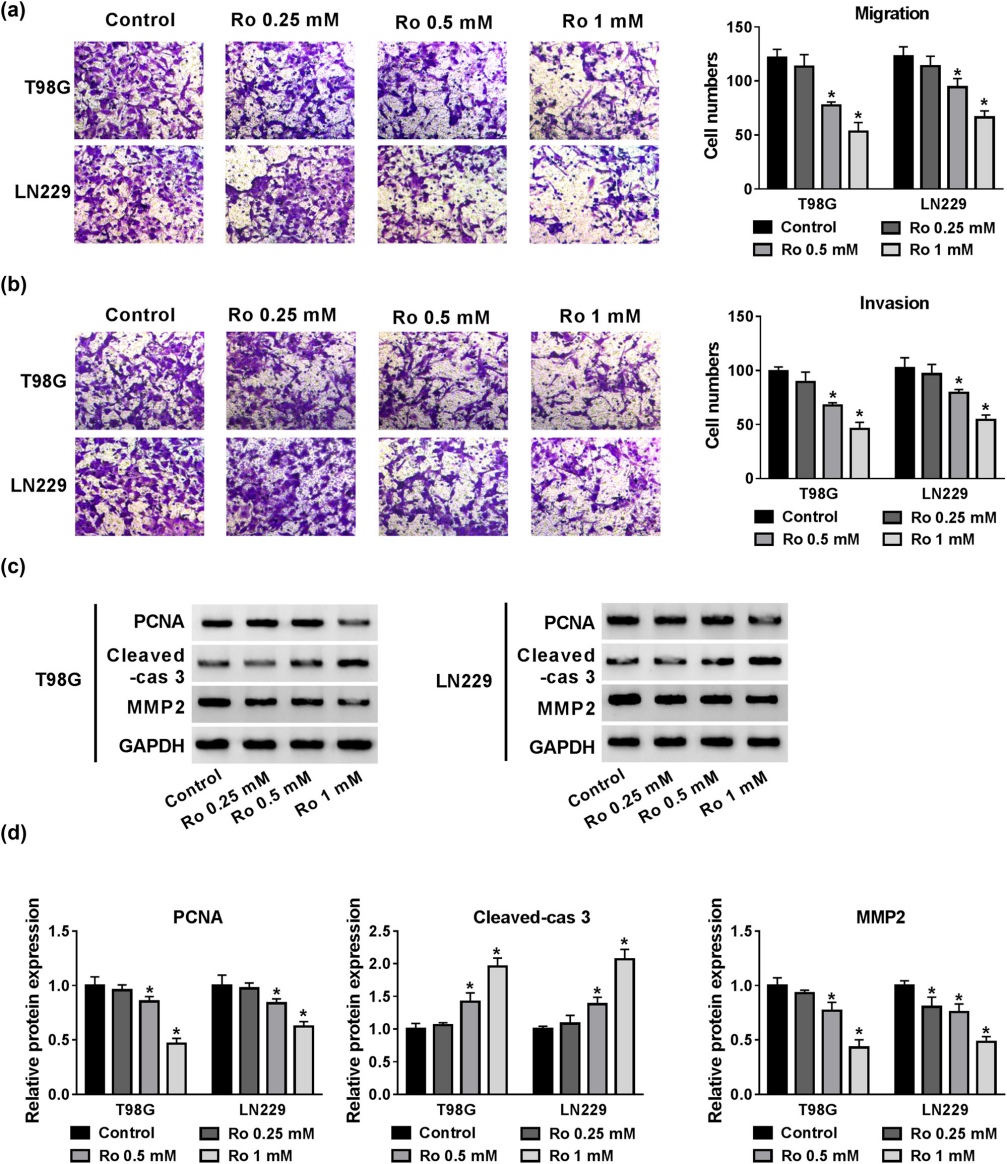
The effect of different concentrations of ropivacaine on migration and invasion of glioma cells. (a and b) Transwell migration and invasion assays were employed to examine the migration and invasion abilities of T98G and LN229 cells treated with ropivacaine. (c and d) The protein expression levels of PCNA, Cleaved-cas 3, and MMP-2 were evaluated by a western blot assay in T98G and LN229 cells treated with ropivacaine. *P < 0.05.

The above results confirmed that ropivacaine had the capability to inhibit proliferation and induce apoptosis of glioma cells. We also explored the effects of ropivacaine on glioma cell migration and invasion using the transwell assay. The results suggested that the cell numbers of migration and invasion declined in T98G and LN229 cells treated with ropivacaine at concentrations of 0.5 and 1 mM compared with the control group (Figure 2a and b). Consistently, apoptosis-related protein Cleaved-cas 3 increased while PCNA and MMP-2 decreased in T98G and LN229 cells after treatment with ropivacaine (Figure 2c and d). Therefore, these results imply that the ropivacaine functioned to suppress the effects of migration and invasion of glioma cells.

### 3.3 Overexpression of SNHG16 overturned the effect of ropivacaine on proliferation, apoptosis, migration, and invasion of glioma cells

**Figure 3:**
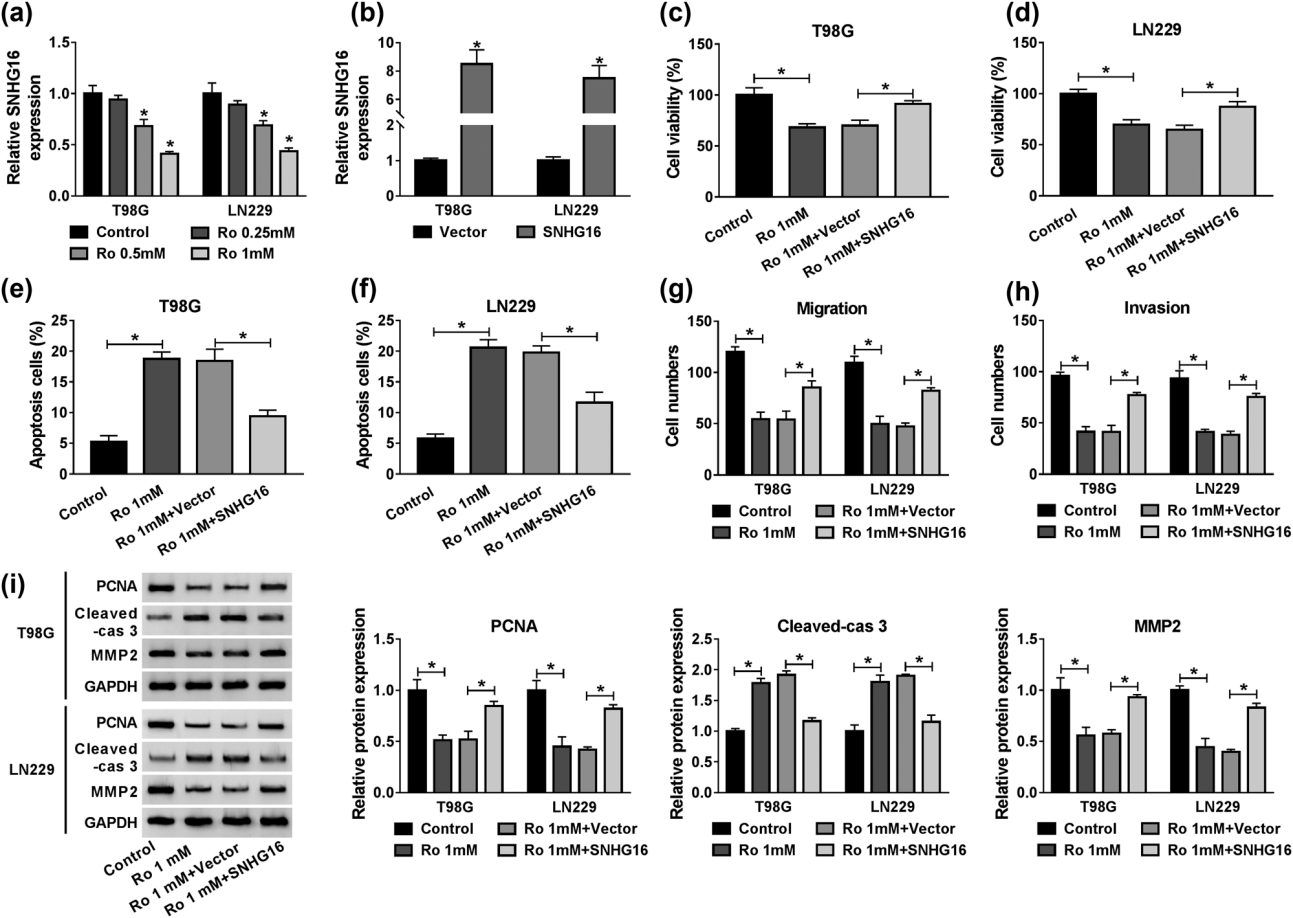
Ropivacaine modulated SNHG16 expression to regulate proliferation, apoptosis, migration, and invasion of glioma cells. (a) Ropivacaine impeded SNHG16 expression in T98G and LN229 cells in a concentration-dependent manner. (b) The expression level of SNHG16 was measured by RT-qPCR in T98G and LN229 cells transfected with Vector or SNHG16. (c–i) T98G and LN229 cells were divided into four groups: control, Ro 1 mM, Ro 1 mM + Vector, and Ro 1 mM + SNHG16. (c and d) The proliferation capability of T98G and LN229 cells was measured using an MTT assay. (e and f) Cell apoptosis rate was measured using a flow cytometry assay in T98G and LN229 cells. (g and h) The transwell assay was applied to assess the migration and invasion of T98G and LN229 cells. (i) Western blot was used to quantify protein expression levels of PCNA, Cleaved-cas 3, and MMP-2 in T98G and LN229 cells. *P < 0.05.

RT-qPCR showed that ropivacaine treatment resulted in the downregulation of SNHG16 in T98G and LN229 cells (Figure 3a). Subsequently, T98G and LN229 cells were transfected with SNHG16 to enhance SNHG16 expression. Non-transfected cells were used as a control. The RT-qPCR results indicated that SNHG16 was elevated by approximately 8-fold in the SNHG16 group compared with the control group (Figure 3b). As shown in Figure 3c and d, the MTT assay showed that overexpression of SNHG16 limited the inhibitory effect of ropivacaine on glioma cell proliferation. Moreover, ropivacaine obviously enhanced cell apoptosis in T98G and LN229 cells, but this was mitigated in the overexpressed vector of SNHG16 (Figure 3e and f). Conversely, when compared with the control, 1 mM of ropivacaine notably decreased cell migration and invasion, which was abolished by upregulation of SNHG16 expression (Figure 3g and h). Consistent with proliferation and apoptosis analyses, ropivacaine induced the upregulation of Cleaved-cas 3, along with the downregulation of PCNA and MMP-2 in T98G and LN229 cells, whereas these effects were reversed by overexpression of SNHG16 in T98G and LN229 cells (Figure 3i). Collectively, ropivacaine impeded proliferation, migration, and invasion, as well as induced apoptosis of glioma cells through downregulation of SNHG16 expression.

### 3.4 MiR-424-5p functioned as a target gene for SNHG16 in glioma cells

**Figure 4:**
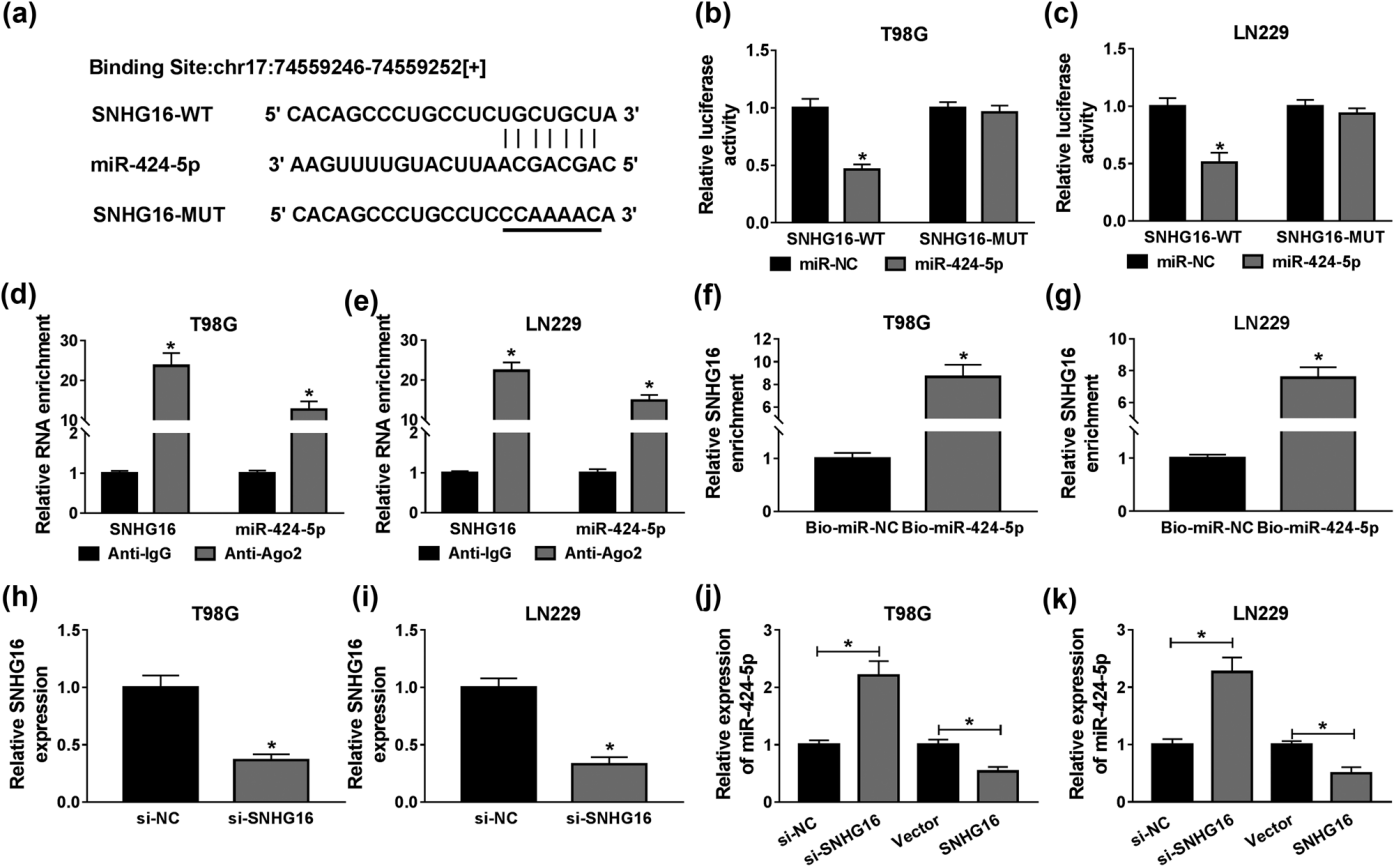
MiR-424-5p was a direct target of SNHG16 in glioma cells. (a) The potential binding sequences between miR-424-5p and SNHG16 are shown. (b and c) The luciferase activity in T98G and LN229 cells was estimated by dual-luciferase reporter analysis. (d and e) The enrichment of miR-424-5p and SNHG16 was calculated by RT-qPCR in each group after RIP analysis. (f and g) An RNA pull-down assay was used to examine the relationship between miR-424-5p and SNHG16 in T98G and LN229 cells. (h and i) Knockdown efficiency of si-SNHG16 was checked in T98G and LN229 cells using RT-qPCR. (j and k) The expression level of miR-424-5p was evaluated by RT-qPCR in T98G and LN229 cells transfected with si-NC, si-SNHG16, Vector, or SNHG16. *P < 0.05.

To identify a possible target gene of SNHG16, starBase tool was used to search for and predict complementary sequences. As shown in Figure 4a, miR-424-5p had complementary sequences in SNHG16. The luciferase activity in T98G and LN229 cells was remarkably decreased in the SNHG16-WT group compared with the control, while no difference was detected in the SNHG16-MUT group, suggesting that miR-424-5p could bind to SNHG16 (Figure 4b and c). After RIP assay, both miR-424-5p and SNHG16 were enriched in the Anti-Ago2 group compared with Anti-IgG group (Figure 4d and e). In addition, the expression level of SNHG16 was drastically higher in the Bio-miR-424-5p group compared to the Bio-miR-NC group (Figure 4f and g). We also observed that SNHG16 was downregulated in T98G and LN229 cells by si-SNHG16 treatment (Figure 4h and i). Interestingly, knockdown of SNHG16 significantly increased the expression of miR-424-5p, and the opposite results were observed in T98G and LN229 cells transfected with overexpressed vector of SNHG16 (Figure 4j and k). In summary, SNHG16 regulated miR-424-5p expression in T98G and LN229 cells in a negative manner.

### 3.5 Silencing of miR-424-5p eliminated effects of ropivacaine on proliferation, apoptosis, migration, and invasion of glioma cells

**Figure 5:**
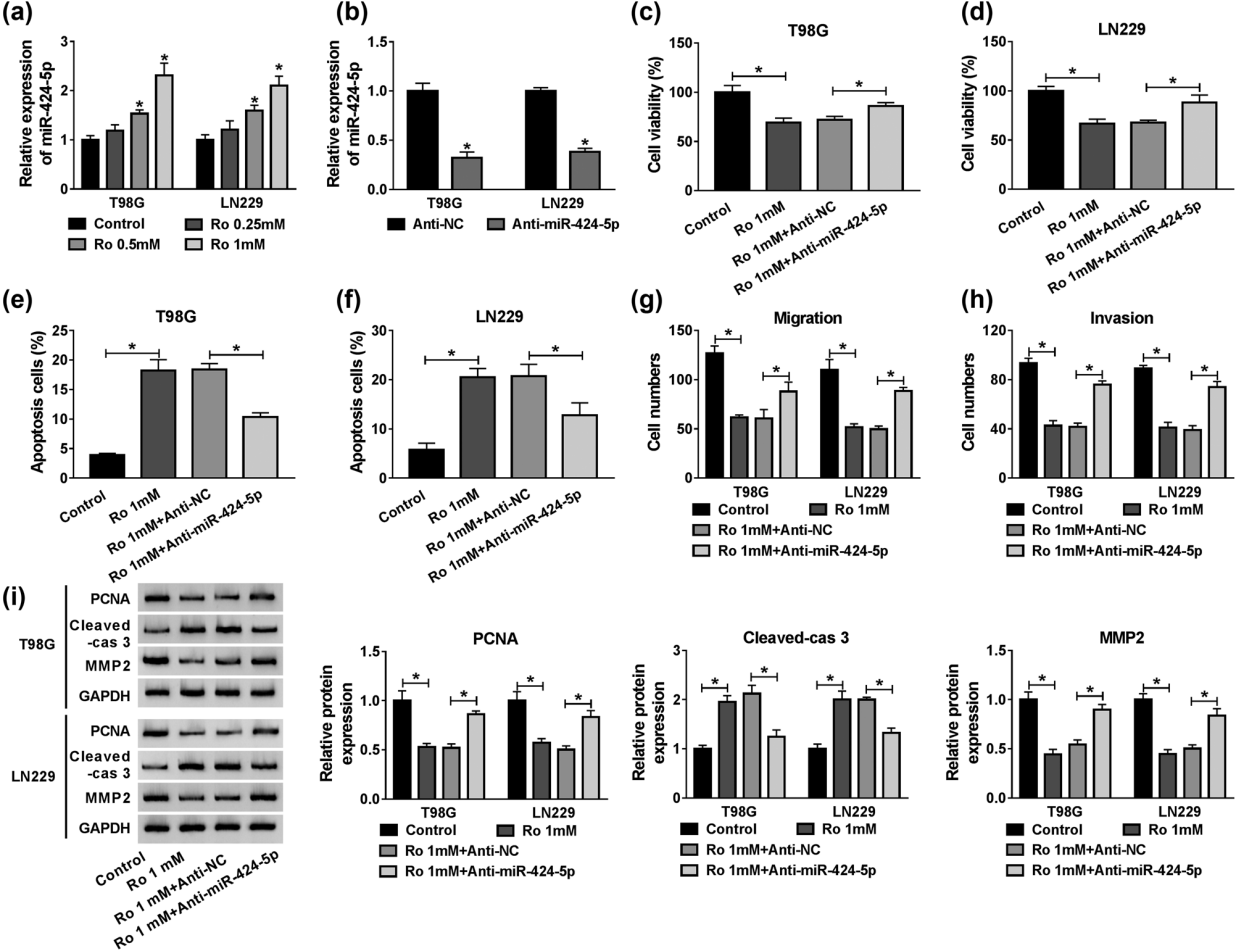
Knockdown of miR-424-5p abolished the effects of ropivacaine on proliferation, apoptosis, migration, and invasion of glioma cells. (a) Ropivacaine enhanced miR-424-5p expression in T98G and LN229 cells. (b) RT-qPCR was used to confirm the knockdown efficiency of Anti-miR-424-5p in T98G and LN229 cells. (c–i) T98G and LN229 cells were treated with Ro 1 mM, Ro 1 mM + Anti-NC, or Ro 1 mM + Anti-miR-424-5p, with untreated cells as control. (c and d) An MTT assay was conducted to measure the cell viability of T98G and LN229 cells. (e and f) The apoptosis rate of T98G and LN229 cells was calculated by flow cytometry. (g and h) Cell migration and invasion assays were performed in T98G and LN229 cells. (i) The protein expression levels of PCNA, Cleaved-cas 3, and MMP-2 were assessed using a western blot assay in T98G and LN229 cells. *P < 0.05.

To investigate whether ropivacaine mediated miR-424-5p expression in T98G and LN229 cells, functional experiments were conducted. As described in Figure 5a, ropivacaine increased miR-424-5p expression in T98G and LN229 cells in a concentration-dependent manner. Moreover, when compared with the Anti-NC group, miR-424-5p decreased in the Anti-miR-424-5p group (Figure 5b). Transfection of Anti-miR-424-5p could cancel out the inhibitory effect on proliferation of T98G and LN229 cells caused by 1 mM of ropivacaine (Figure 5c and d). As presented in Figure 5e and f, downregulation of miR-424-5p protected T98G and LN229 cells from ropivacaine-induced apoptosis. Besides, the loss of migration and invasion abilities induced by ropivacaine was reinstated by silencing miR-424-5p (Figure 5g and h). Furthermore, ropivacaine led to the overexpression of Cleaved-cas 3 and repression of PCNA and MMP-2, yet these effects could be remarkably abolished by knockdown of miR-424-5p in T98G and LN229 cells (Figure 5i). The above results revealed that miR-424-5p silencing could partially rescue ropivacaine induced effects on proliferation, apoptosis, migration, and invasion of glioma cells.

### 3.6 Ropivacaine regulated proliferation, apoptosis, migration, and invasion of glioma cells by targeting SNHG16/miR-424-5p axis

**Figure 6:**
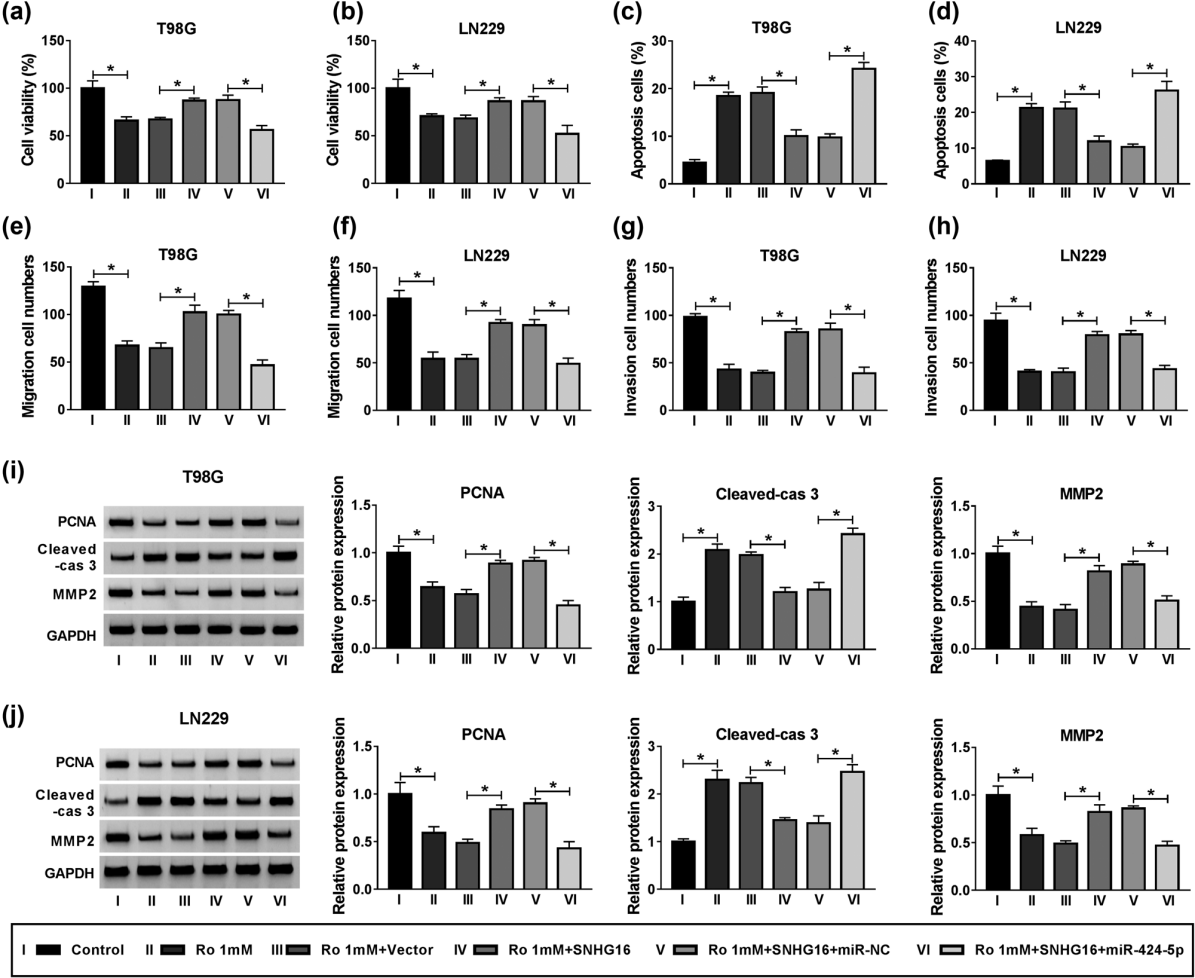
Overexpression of miR-424-5p abrogated the effects of SNHG16 overexpression on proliferation, apoptosis, migration, and invasion of glioma cells treated with ropivacaine. (a–j) T98G and LN229 cells were divided into six groups: control, Ro 1 mM, Ro 1 mM + Vector, Ro 1 mM + SNHG16, Ro 1 mM + SNHG16 + miR-NC, and Ro 1 mM + SNHG16 + miR-424-5p. (a and b) The MTT assay was introduced to show the cell activity of T98G and LN229 cells after transfection. (c and d) The flow cytometry assay was used to assess apoptosis of T98G and LN229 cells. (e–h) Cell migration and invasion capability were examined with transwell analysis in T98G and LN229 cells. (i and j) The western blot assay was applied to evaluate the protein expression level in T98G and LN229 cells. *P < 0.05.

To determine the association between SNHG16 and miR-424-5p in ropivacaine-induced glioma cells, T98G and LN229 cells were transfected with a vector, SNHG16, SNHG16 + miR-NC, or SNHG16 + miR-424-5p. Under ropivacaine conditions, the results of the MTT assay indicated that co-transfection of SNHG16 and miR-424-5p could counteract the SNHG16-induced upregulation of cell activity (Figure 6a and b). The flow cytometry assay demonstrated that overexpression of SNHG16 inhibited cell apoptosis, which was abolished by upregulation of miR-424-5p in T98G and LN229 cells treated with ropivacaine (Figure 6c and d). Besides, overexpression of SNHG16 increased the migration and invasion of glioma cells treated with ropivacaine, while these enhancement effects were abolished by transfecting with miR-424-5p mimic (Figure 6e–h). Under ropivacaine conditions, overexpression of miR-424-5p abrogated the inhibitory effect on Cleaved-cas 3 expression and the enhancement effects on PCNA and MMP-2 expression in T98G and LN229 cells, caused by overexpression of SNHG16 (Figure 6i and j). Taken together, by regulating the SNHG16/miR-424-5p axis, ropivacaine promoted apoptosis while suppressing proliferation, migration, and invasion of glioma cells.

## 4 Discussion

Collectively, our results indicate that ropivacaine repressed the viability of glioma cells in a dose/time-dependent manner. Furthermore, we found that ropivacaine was capable of inhibiting migration and invasion, as well as enhancing apoptosis of glioma cells. Mechanistically, overexpression of SNHG16 attenuated the anti-tumor effects of ropivacaine in glioma by regulating miR-424-5p. Ropivacaine is regarded as an amide-linked local anesthetic for the relief of pain in nerve blocks, interventional spinal procedures, and tumor removal [18,19]. Moreover, *in vitro* and *in vivo* studies proved that local anesthetics might also have direct effects on certain tumor cells, including non-small cell lung cancer [20], hepatocarcinoma cells [21], and thyroid cancer cells [22]. Recently, Li et al. implied that exposure to ropivacaine could prompt cytotoxicity in UCMSCs by impeding cell growth, regulating the cell cycle, and stimulating apoptosis [23]. Wang et al. revealed that ropivacaine stimulated apoptosis of hepatocellular carcinoma cells through interaction with caspase-3 [24]. The caspase-3 was a crucial component that correlated with the apoptotic signaling pathway [25]. Similarly, our results indicate that ropivacaine inhibits glioma progress by suppressing proliferation, migration, and invasion while inducing apoptosis of glioma cells.

Furthermore, the crosstalk between lncRNAs and miRNAs plays a critical role in biological processes, including the initiation and progression of glioma [26]. LncRNA SNHG16 could directly target miR-424-5p and inhibit its expression in glioma cells. The novel mode of regulation of lncRNA-miRNA has been widely recognized in recent research [27]. The results of the RT-qPCR assay show that miR-424-5p was upregulated by silencing of SNHG16 in glioma cells. Zhu et al. confirmed that SNHG16 is enriched in cervical cancer tissues and cells, relative to normal controls [28]. Analogously, a report of Yu et al. revealed that SNHG16 played a tumorigenic role in glioma [29]. Not surprisingly, our data confirmed that the lncRNA SNHG16 functions as an oncogene, contributing to glioma progression by acting as miR-424-5p sponge.

Our results imply that silencing of miR-424-5p abolishes the suppression effects on glioma proliferation, migration, and invasion caused by ropivacaine. In agreement with a previous study [30], our results suggest that miR-424-5p has a carcinoma suppressor role in the development of glioma. Similar results were also documented in cervical and renal cancers, whereby miR-424 exerted its tumor inhibitor roles by impeding cell growth and increasing apoptosis [31,32]. Previous studies suggest that miRNA serves as an essential regulator in mediating target gene expression [33]. Further work should be undertaken to investigate the SNHG16/miR-424-5p axis and the possible target mRNAs of miR-424-5p.

Collectively, the data generated in this study reveal that ropivacaine induces apoptosis and has an inhibitory effect on glioma cell growth and invasion. Notably, our findings demonstrate that ropivacaine inhibits expression of SNHG16, while enhancing the expression of miR-424-5p in glioma cells. Under ropivacaine stimulating conditions, upregulation of miR-424-5p inverted the effects of SNHG16 overexpression on glioma cells. These findings indicate that the SNHG16/miR-424-5p axis in combination with ropivacaine might be an effective treatment strategy against glioma in the future.

## 5 Conclusion

The current study revealed that ropivacaine can inhibit proliferation, migration, and invasion while inducing apoptosis of glioma cells *in vitro*. Mechanistically, ropivacaine exerted its carcinoma inhibition by regulating the SNHG16/miR-424-5p signal pathway, which provides a reference for future studies on glioma treatment.
